# Supplementary material for: Experimental investigation of the influence of friction, surface roughness and material hardness on the external load factor in threaded joints
Source: PLoS One. 2026 Jan 5;21(1):e0340073. doi: 10.1371/journal.pone.0340073 (PMC12768235; doi:10.1371/journal.pone.0340073)
Supplement: Attachment — Submitted filename: Review Comments to the Author.pdf [file pone.0340073.s002.pdf]

Review Comments to the Author (Reviewer 1)

The manuscript presents a clear and well-structured experimental study aimed at evaluating how friction coefficient, surface roughness, and material hardness influence the external load factor (χ) in bolted joints. The topic is relevant for structural and mechanical engineering, and the experimental approach is sound. The text is well-organized, and the theoretical background is adequately introduced.

The description of the forces and how they are transmitted is not sufficiently clear. In particular, the forces and moments Fb, Mb, and Fm, Mm, are introduced but not adequately contextualized. I would suggest elaborating on them and, if appropriate, including them in the schematic of Figure 1 for better clarity.

In general, the figure captions should be more descriptive to make the interpretation of what is being shown more immediate.

Regarding Figure 6, the residuals do not appear to be normally distributed as stated in the text. This point should be justified and corrected. While the normal probability plot does suggest that a single variable is sufficient to describe the observed data, there is a visible bias in the residuals for lower values, as shown in the "Versus Fit" plot. Please justify this result. Moreover, this plot should be renamed to something more explanatory.

Figure 8 does not add new information and appears to be confusing. I would suggest removing it. The same for figure 11.
