# Supplementary material for: Experimental investigation of the influence of friction, surface roughness and material hardness on the external load factor in threaded joints
Source: PLoS One. 2026 Jan 5;21(1):e0340073. doi: 10.1371/journal.pone.0340073 (PMC12768235; doi:10.1371/journal.pone.0340073)
Supplement: Attachment — Submitted filename: Response to Reviewers.docx [file pone.0340073.s004.docx]

**Experimental Investigation of the Influence of Friction, Surface Roughness and Material Hardness on the External Load Factor in Threaded Joints**

Dear Editor and Reviewers,

The revised manuscript has been resubmitted. All modifications have been highlighted in red for easy reference. We truly appreciate your valuable comments and support during the preparation of the paper.

**1. Detailed Revisions in Response to Reviewer #1’s Comments**

**Reviewer #1:** The manuscript presents a clear and well-structured experimental study aimed at evaluating how friction coefficient, surface roughness, and material hardness influence the external load factor (χ) in bolted joints. The topic is relevant for structural and mechanical engineering, and the experimental approach is sound. The text is well-organized, and the theoretical background is adequately introduced.

**1. Comment No. 1:** The description of the forces and how they are transmitted is not sufficiently clear. In particular, the forces and moments Fb, Mb, and Fm, Mm, are introduced but not adequately contextualized. I would suggest elaborating on them and, if appropriate, including them in the schematic of Figure 1 for better clarity.

In general, the figure captions should be more descriptive to make the interpretation of what is being shown more immediate.

**Response to Comment No. 1:**

We sincerely thank the reviewer for the valuable comments regarding Section 2. We have **completely rewritten Section 2** (See the manuscript) to ensure clarity, intuitiveness, and readability. Specifically, we have:

- Provided detailed explanations of all terms, including the “YY plane,” “reference axis,” and centroid point C so that readers can easily understand their positions and roles in the force equilibrium analysis.

- Updated all figures to clearly show all external and internal forces acting on the system, and added the missing force symbols (F_b_, F_m_, etc.) corresponding to the equations presented.

- Thoroughly checked all equations and force diagrams to ensure their accuracy and consistency with the theoretical analysis.

These revisions make Section 2 more accessible, accurate, and intuitive, allowing readers to fully understand the content without ambiguity.

**2. Comment No. 2:** Regarding Figure 6, the residuals do not appear to be normally distributed as stated in the text. This point should be justified and corrected. While the normal probability plot does suggest that a single variable is sufficient to describe the observed data, there is a visible bias in the residuals for lower values, as shown in the “Versus Fit” plot. Please justify this result. Moreover, this plot should be renamed to something more explanatory.

**Response to Comment No. 2:**

We thank the reviewer for the comment regarding Figure 6. After a more detailed examination, we acknowledge that the residuals exhibit a slight deviation at low fitted values, particularly in the Residuals versus Fitted Values plot. However, this deviation does not compromise the validity of the model for the following reasons:

**(1) On the normality of residuals:** In a second-order RSM model, minor deviations near the boundaries of the design space are common due to localized nonlinear behavior. Nevertheless, as shown in the Normal Probability Plot, the residuals follow an almost linear trend without curvature or abnormal dispersion, indicating that the normality assumption is sufficiently satisfied for ANOVA and regression diagnostics.

**(2) On the deviation at the lower fitted region in the “Versus Fit” plot:** The slight deviation observed at low fitted values arises from the high concentration of experimental points in the region of low μ, where sensitivity is higher and local errors tend to be more pronounced. More importantly, neither the Residuals versus Observation Order plot nor the histogram reveals any systematic pattern, meaning there is no evidence of heteroscedasticity or autocorrelation. Therefore, the model still meets the assumptions of independence and constant variance of the residuals.

**(3) On the overall adequacy of the model:** The global model statistics R² = 99.96%, R²_Adj_ = 99.93% and R²_Pred_ = 99.94%, demonstrate that these minor residual deviations do not affect the predictive capability or the overall quality of the regression model. These values indicate an excellent level of fit consistent with standard RSM/DOE practices.

**(4) On renaming the plot as requested by the reviewer:** Following the reviewer’s suggestion, we have renamed the plot from:

- “Versus Fit” to: **“Residuals Versus Fitted Values”,**

- “Versus Oder” to: **“Residuals Versus Observation Order”**

This new title is more technically accurate and clearer for readers.

We have updated the figure caption, axis labels, and the corresponding explanation in the manuscript to clarify the behavior of the residuals and reinforce the adequacy of the proposed model.

We have rewritten “Section 4.3.2”:

**4.3.2 Evaluation of residuals in the regression model**

Figure 6 presents the diagnostic plots used to assess the residual behavior of the regression model. The normal probability plot shows that the residuals follow an approximately linear trend, indicating that the normality assumption is reasonably satisfied. The small deviation at the lower tail is typical for second-order RSM models and does not represent a systematic pattern.

In the residuals versus fitted values plot, slight deviations are observed at low fitted levels due to the concentration of experiments at low μ; however, no funnel shape or variance trend is present, confirming that the assumption of constant variance holds. The residuals versus observation order plot also shows no noticeable trend or cyclic behavior, demonstrating that the residuals are independent.

Fig 6. Diagnostic plots for evaluating the residual behavior of the regression model.

Overall, the diagnostic plots confirm that the residuals are randomly scattered without any systematic patterns, and the model satisfies the key assumptions of normality, independence, and homoscedasticity. These results validate the use of the second-order RSM model for prediction, significance analysis, and optimization of the external load factor (χ) within the investigated design space. Consequently, the model is considered reliable for evaluating the influence of technological parameters including surface roughness, friction coefficient and material hardness on load distribution behavior in bolted joints.

**3. Comment No. 3:** Figure 8 does not add new information and appears to be confusing. I would suggest removing it. The same for figure 11.

**Response to Comment No. 3:**

We thank the reviewer for this suggestion. **Figure 8 has been removed** due to redundancy. However, we believe Figure 11 should be retained because it provides unique information on the convergence behavior and robustness of the optimization process, which cannot be conveyed by a numerical table alone. To make this clearer, we have revised the caption and expanded the explanation in Section 4.6.2 We hope the reviewer agrees that keeping Figure 11 enhances the transparency and interpretability of the optimization results.

We have rewritten “Section 4.6.2”:

**4.6.2 The optimal values of the input factors**

The optimization procedure was performed using the desirability-based optimization approach to identify the combination of μ(A), Ra(B), and HB(C) that produces the target external load factor χ(y) ≈ 0.25. The optimal solution obtained from the response surface model corresponds to μ = 0.1809, Ra = 3.5181 µm and HB = 216.859, yielding a predicted value of χ ≈ 0.2501. The composite desirability reaches 0.9996, demonstrating an excellent agreement between the optimization objective and the model prediction, and thus confirming the reliability of the regression model within the design domain.

Figure 10 illustrates the convergence behavior of the optimization process, including the evolution of the predicted χ, the desirability function, and the iterative adjustment of μ, Ra, and HB. This visualization demonstrates that the optimum is achieved through a stable and monotonic convergence path, confirming that the final solution is robust and not highly sensitive to initial conditions. The coordinated adjustment of the three input factors further highlights the nonlinear interaction effects identified in the regression analysis.

Overall, the optimization results establish that the target value χ ≈ 0.25 can be achieved with high confidence, and they validate the practical applicability of the developed regression model for guiding the selection of technological parameters in threaded joint assemblies.

**Fig 10.** **Convergence behavior of the response surface optimization process.**

**2. Detailed Revisions in Response to Reviewer #2’s Comments**

**Reviewer #2:** This study experimentally investigates how friction coefficient, surface roughness, and material hardness of clamped plates influence the external load factor (χ) in threaded joints. Results show that material hardness has the strongest effect, with χ decreasing as hardness increases, while surface roughness has a nonlinear impact and friction coefficient also plays a significant role. A regression model developed using the Box–Wilson method accurately predicts χ, with an optimization identifying the best parameter combination for reliable joint performance. The findings provide practical insights for improving the design and reliability of bolted joints in engineering applications. While the paper appears to contribute meaningfully to the state of the art, the overall integrity of the manuscript is questionable. The text is difficult to follow, and the terminology used is challenging for readers, even for experts in the field. I believe a comprehensive revision of both the text and structure (potentially a polished, rewritten version) is necessary before the manuscript can be considered for possible publication. The main points for improvement are as follows:

**1. Comment No. 1:** The Abstract and Introduction are reasonably well-prepared. However, the content in Section 2 is unclear, and many terms are vaguely defined. For example, it is not evident what is meant by the “YY plane” (is this the 2D plane where equilibrium has been illustrated in Fig. 1?), or what is referred to as the “reference axis” (is this the trimmed line?). Similarly, the centroid point O, introduced in another section cut, needs clarification regarding its relation to the upper section (is it a vertical section from the Y-direction main vector plane?). All figures should be revised to provide sufficient detail for comprehension rather than requiring the reader to decode them.

**Comment No. 2:** It is strongly advised that the authors present all external and internal forces acting on the system to improve clarity. The terms defined in Eq. (1) are not visible in Fig. 1, making their definition and relevance to the study ambiguous. Definitions of variables such as Fb and Fm1 are also missing. Section 2 must be rewritten with adequate explanations of the equations and free-body equilibrium diagrams to ensure reader understanding.

**Response to Comment No. 1 and 2:**

We sincerely thank the reviewer for the valuable comments regarding Section 2. We have **completely rewritten Section 2** (See the manuscript) to ensure clarity, intuitiveness, and readability. Specifically, we have:

- Provided detailed explanations of all terms, including the “YY plane,” “reference axis,” and centroid point C, so that readers can easily understand their positions and roles in the force equilibrium analysis.

- Updated all figures to clearly show all external and internal forces acting on the system, and added the missing force symbols (F_b_, F_m_, etc.) corresponding to the equations presented.

- Thoroughly checked all equations and force diagrams to ensure their accuracy and consistency with the theoretical analysis.

These revisions make Section 2 more accessible, accurate, and intuitive, allowing readers to fully understand the content without ambiguity.

**Comment No. 3:** In Section 3, the study seems to describe a system designed for a special purpose based on mathematical assumptions. The order of Figs. 1, 2, and 3 should be revised, with corresponding explanations currently in Section 3 moved to Section 2.

**Response to Comment No. 3:**

We sincerely thank the reviewer for this valuable comment. Following your recommendation, the conceptual explanation of the measurement principle, originally placed at the beginning of section 3, has been relocated to the end of section 2, immediately after the theoretical formulation of the external load factor (Equations (20) and (21)). We have also rewritten Section 3.1. This improves the logical continuity between the theoretical background and the experimental methodology.

In addition, the figures have been reorganized to reflect the logical progression of the study. Figure 2, “Force and stress distributions in a bolted joint,” belongs to the theoretical framework and illustrates the load transfer mechanism; therefore, it has been added to Section 2. The updated figure sequence is as follows:

**- Figure 1:** Bolted joint load model (theoretical framework)

**- Figure 2:** Force and stress distributions in a bolted joint (theoretical framework)

**- Figure 3:** Schematic diagram of the measurement system (principle of operation)

**- Figure 4:** Experimental setup (actual apparatus)

**- Figure 5:** Installation of the donut loadcell (sensor configuration)

Corresponding figure citations and section references throughout the manuscript have been updated accordingly.

We appreciate the reviewer’s constructive feedback, which has significantly improved the clarity and structure of the manuscript.

We have rewritten “Section 3.1”:

**3.1 Experimental setup**

The experimental setup for determining the external force coefficient is shown in Fig 4.

*1. External force display meter; 2. Electrical control cabinet; 3. Bolt tightening force display meter; 4. Hydraulic system; 5,11. Clamping plates; 6. Support frame; 7. Force applicator arm; 8. Tilt angle adjuster (α); 9. Bolt; 10. Loadcell measuring bolt tightening force*

**Fig 4.** **Experimental Setup**

The test specimen consists of two steel plates joined by an M16 bolt through a circular hole, subjected to axial loading from the hydraulic cylinder. Two types of loadcells are used: (i) S-type loadcell (Zemic B3G, 0–20 kN, stainless steel) measures the external load; (ii) Donut through-hole loadcell (Forsentek FBDA, 0–20 kN) measures the bolt tightening force.

The sensors have received factory calibration with full-scale accuracy ±0.05%, repeatability ±0.02%, and linearity error below ±0.1%, ensuring reliable and stable measurements throughout the loading process.

The configuration and installation method of the donut-type loadcell are illustrated in Fig 5. In the technical diagrams (Figs 4 and 5), geometric dimensions are presented in millimeters (mm), while force measurements are expressed in kilonewtons (kN). The sensor outputs are transmitted to a data acquisition system and displayed through a force indicator, enabling continuous recording of variations in bolt tightening force under the applied axial load.

*1. Bolt M16; 2. Loadcell measuring bolt tightening force; 3,4. Clamping plates*

**Fig 5.** **Installation of donut loadcell for bolt preload measurement**

**Comment No. 4:** Section 3.2 suggests that surface roughness directly relates to friction. If this is the case, the two factors cannot be considered independent variables. The authors need to justify why both were included in their analysis and equations.

**Response to Comment No. 4:**

We appreciate the reviewer’s insightful comment. Although surface roughness (Ra) and friction coefficient (μ) are physically related, in this study they are treated as controllable and distinguishable engineering factors, and therefore can be considered independent variables within the DOE framework.

Specifically, the friction coefficient μ in bolted joints is not determined solely by surface roughness; rather, it is strongly influenced by lubrication conditions, contamination, wear state, and surface chemistry. In our experiments, μ was intentionally controlled through the use of lubricated vs. unlubricated conditions in accordance with ISO 16047. As a result, μ could vary independently of the machined surface roughness.

In contrast, Ra represents the geometric texture of the contact surfaces, which affects the real contact area and local deformation during tightening. Ra was controlled by machining processes (finely machined vs. rough surfaces), independent of the lubrication condition used to set μ. These two control actions do not overlap and thus allow the experimental design to isolate the geometric effect (Ra) from the tribological condition (μ).

Therefore, including both μ and Ra in the regression model is necessary to (i) examine their individual contributions to the external load factor χ and (ii) capture potential interaction effects between surface geometry and lubrication-related friction. This distinction is consistent with DOE principles and with practices in tribology research, where friction and surface roughness are often varied independently to evaluate their separate influences.

We have added a clarification in Section 3.2 to explicitly explain the independence of the two factors.

we have added the following content to Section 3.2:

Although Ra may influence frictional behaviors, μ and Ra were controlled through different physical mechanisms in this study. Ra was adjusted solely through machining processes, whereas μ was adjusted through lubrication state. Friction in bolted joints depends on several factors beyond surface geometry, such as lubricant thickness, surface chemistry, and contamination, meaning that μ cannot be predicted from Ra alone. Consequently, μ and Ra are treated as independent variables in accordance with DOE principles, enabling: independent quantification of geometric (Ra) and tribological (μ) effects, identification of interaction effects between Ra and μ, and a more comprehensive understanding of how assembly conditions influence χ.

**Comment No. 5:** The quality of figures and tables must be improved throughout the manuscript. Figure 5 appears redundant as it repeats content already shown elsewhere, with little justification for its re-presentation. Descriptions within figures and in the main text are insufficient, making interpretation difficult and undermining the paper’s integrity and quality. Additionally, if no “Note” exists in Table 4, it should be removed.

**Response to Comment No. 5:**

We thank the reviewer for the suggestions regarding the figures and tables. Specifically:

**- Figures’ Quality:** All figures have been enhanced with higher resolution and improved clarity to ensure readability. Captions have been revised to provide more detailed explanations, facilitating easier interpretation of the data.

**- Figure 5:** We acknowledge that Figure 5 duplicated information already presented in other figures. To address this, we have **removed Figure 5** to avoid redundancy.

**- Table 4:** The “Note” section has been removed from Table 4 since no additional clarification was necessary, in accordance with the reviewer’s suggestion.

These changes improve the overall presentation and integrity of the manuscript.

**Comment No. 6:** A clear explanation is required on how the dataset for Fig. 7 was obtained, how the data were summarized in Table 5, and whether the results were derived experimentally, numerically, or through another approach. The rationale for applying linear equations without considering dimensional analysis or the Buckingham π-theorem is unclear. The manuscript must also explain whether the Box–Wilson method adequately satisfies these theoretical requirements.

**Response to Comment No. 6:**

Thank you for the valuable comment. The data used in Figure 7 were obtained directly from physical experiments performed according to the Box–Wilson design. All χ values were measured using the load-cell system described in Section 3. The results in Table 5 are statistical outputs (ANOVA, lack-of-fit, and model significance) generated from these experimental data using Minitab; no numerical simulation was applied.

The regression equations are based on the Box–Wilson response surface methodology, in which all variables are first converted into coded, dimensionless values. Therefore, applying linear and quadratic terms does not require dimensional analysis or the Buckingham π-theorem. RSM inherently satisfies these requirements through its coded-variable system and polynomial approximation structure. The model adequacy is confirmed by the ANOVA results (F = 2976.57, P = 0.0) and the nonsignificant lack-of-fit (P = 1.0), demonstrating that the Box–Wilson method is theoretically appropriate and statistically reliable for this study.

**Comment No. 7:** The final paragraph of the Conclusion does not present conclusions but rather a potential application of the study; it should either be moved to another section or removed entirely. Moreover, the regression results and the effects of each factor, discussed earlier in the paper, are missing from the Conclusion. The most significant achievement appears to be the relationships among the factors, their percentage contributions, and the coefficients obtained from the Box–Wilson method, and this should be emphasized.

**Response to Comment No. 7:**

We sincerely thank the reviewer for this insightful comment. Following your recommendation, the final paragraph of the Conclusion (Previously describing potential applications) has been removed from this section and incorporated into the “Future Research Directions” subsection, ensuring that the Conclusion contains only the key scientific findings of the study.

Additionally, the Conclusion has been substantially revised to explicitly highlight the core contributions of the work, including:

- The quantitative relationships among friction coefficient, surface roughness, and material hardness obtained from the Box–Wilson regression model,

- The statistically significant regression coefficients that form the final predictive equation,

- The nonlinear effects and interactions among factors (μ, Ra, HB),

- The relative influence of each parameter on the external load factor χ.

The revised Conclusion now synthesizes the major findings from sections 4.1 to 4.6, emphasizing that material hardness contributes the greatest effect on χ, followed by surface roughness and friction, consistent with the regression significance tests and effect plots.

These modifications ensure that the Conclusion accurately reflects the scientific contributions of the study and aligns with the structural expectations of high-quality journals.

We have rewritten the “Conclusion”:

This study conducted an experimental analysis to evaluate the effects of three technological factors, including combined coefficient of friction, surface roughness, and material hardness of the clamped plate on the external load factor (χ) of threaded joints. A second-order regression model was developed based on a Rotatable Central Composite Design (Box–Wilson) combined with a dedicated measurement system, enabling a quantitative description of the nonlinear behavior and interaction effects among these parameters.

The statistical analysis confirmed that all linear, quadratic, and interaction terms in the model were significant at the 95% confidence level (P < 0.05). The resulting predictive equation exhibited excellent agreement with experimental data (R² = 99.96%; R²_Adj_ = 99.93%; R²_Pred_ = 99.94%), demonstrating the robustness and reliability of the model.

The regression results indicated that friction coefficient (μ) had the strongest influence (linear coefficient +1.06; quadratic coefficient –2.78), producing a nonlinear trend with a maximum at μ ≈ 0.2. Surface roughness (Ra) also exhibited a pronounced nonlinear effect (linear coefficient +0.024; quadratic coefficient –4.14×10⁻³), with a maximum at Ra ≈ 3.9 µm. Material hardness (HB) caused a stable decrease in the external load factor (χ) over the entire range studied (linear coefficient –1.01×10⁻³).

The evaluation of the relative contributions based on the total absolute T-value showed that μ accounted for ≈46%, Ra ≈35%, and HB ≈19% of the variation in the external load factor (χ). The Ra×HB interaction was the strongest among the pairs, indicating a significant dependence of roughness effects on material hardness. These results are consistent with the analyses presented in the discussion and reinforce the comprehensiveness of the regression model.

Response Optimizer predicted χ ≈ 0.2501 at the optimal combination μ = 0.1809, Ra = 3.5181 µm, HB = 216.859, with a total desirability of 0.9996, indicating high predictive and optimization capability. Overall, this study provides a reliable quantitative framework for controlling the external load factor, directly contributing to the enhanced reliability of threaded joints in engineering applications.

**Comment No. 8:** The final paragraph of the Conclusion does not present conclusions but rather a potential application of the study; it should either be moved to another section or removed entirely. Moreover, the regression results and the effects of each factor, discussed earlier in the paper, are missing from the Conclusion. The most significant achievement appears to be the relationships among the factors, their percentage contributions, and the coefficients obtained from the Box–Wilson method, and this should be emphasized.

**Comment No. 8 overlaps with Comment No. 7.**

**Comment No. 9:** Only 9 out of 19 cited references are from the past five years. The literature review should be updated with more recent and relevant works to strengthen the paper’s contribution to the state of the art.

**Response to Comment No. 9:**

We would like to thank the reviewer for this valuable comment. In response, we have updated and expanded the literature review by adding several recent studies (within the past five years) that are directly related to friction, surface roughness, material hardness, and the behavior of threaded joints under external loading. These additions help clarify the current research context and highlight the novelty and contributions of our work to the field of threaded joint analysis. The updated references have been incorporated into Section 1 of the revised manuscript.

Recent studies have been incorporated into the manuscript:

[1]	Li H, Zhao Y, Jiang J, Wang H, He J, Liu J, et al. Effect of frequency on the fatigue performance of bolted joints under axial excitation. Tribology International. 2022;176:107933.

[3]	Zheng M, Li Y, Guo J, Yan X, Liu Z, Wahab MA. A novel theoretical analysis on mechanical properties of bolted joints under torsional loads considering service torque. Engineering Structures. 2025;325:119410.

[4]	Ramsey JK. Mechanics of Preloaded Bolt Tensile Loading With Focus on Load Introduction Factor. National Aeronautics and Space Administration, 2025.

[5]	Wang C, Du M, Zheng M, Sun Q. Study on the stress evolution at threads of bolted joint under transverse loads. Proceedings of the Institution of Mechanical Engineers, Part C: Journal of Mechanical Engineering Science. 2025;239(6):2106-16.

[11]	Li Y, Lang L, editors. Theoretical and experimental study on the damage behavior of bolted joints under cyclic loads. Structures; 2024: Elsevier.

[15]	Qiu P-q, Wang W-w, Wang K, Zhang X-q, Ning J-g, Zhao C-l, et al. Experimental study on the energy dissipation mechanism of bolted rock under dynamic loading. Sci. Rep. 2025;15(1):17182.

[16]	He T, Chen W, Liu Z, Gong Z, Du S, Zhang Y. The Impact of Surface Roughness on the Friction and Wear Performance of GCr15 Bearing Steel. Lubricants. 2025;13(4):187.

[21]	Chen Y, Yamaguchi T, Hayashi G, Yamauchi M, Ueno K. Experimental study on long friction-type bolted joint combined with interference fit bolt. Advances in Bridge Engineering. 2024;5(1):6.

**Comment No. 10:** Finally, while the proposed future directions add some value, they would be more compelling if linked explicitly to the paper’s contribution to the field. For example: Did the study omit certain influential factors identified through Buckingham’s π-theorem and dimensional analysis? Was reliability analysis, as outlined in Section 4, neglected? Was a laboratory-scale benchmark for load testing not designed? Regardless of the answers, the paper must clearly explain how this study differentiates itself from previous research and what unique contribution it offers.

**Response to Comment No. 10:**

We thank the reviewer for the thoughtful comment. Following your suggestions, we have revised the “Future Research Directions” section to explicitly connect the proposed extensions with the core contributions of this study. The issues raised in your questions have been addressed as follows:

**(1) Regarding influential factors identified through Buckingham’s π-theorem and dimensional analysis:**

In the present study, we intentionally focused on three primary and controllable assembly parameters, μ, Ra, and HB as the core factors governing the external load factor χ. Other π-theorem variables (e.g., thread geometry, member stiffness, joint parameters) were not omitted but treated as fixed conditions in the experimental design to isolate the effects of the selected factors.

**(2) Regarding the reliability analysis mentioned in Section 4:**

The reliability-based analysis was not neglected; rather, it was proposed as an extension beyond the scope of the current regression-focused work. We have now clarified that the regression coefficients and quantitative relationships established in this study form the analytical foundation for future RBDO development.

**(3) Regarding the laboratory-scale benchmark for load testing:**

The experimental setup developed in this study was designed specifically to capture the external load factor under controlled conditions. While a broader standardized benchmark was not constructed, we have clarified that the measurement system itself constitutes a significant methodological contribution and will serve as the basis for future benchmark development.

**(4) Regarding the unique contribution and differentiation from previous studies:**

The Conclusion section has been revised to clearly highlight the central contributions of this work:

- Establishing quantitative nonlinear relationships among μ, Ra, HB, and χ;

- Determining the percentage contribution of each factor;

- Providing statistically significant regression coefficients through the Box–Wilson method;

- Identifying the strongest interaction effects;

- Delivering an experimentally validated measurement system.

These elements differentiate the present study from prior research and define its unique contribution to the field.

We appreciate the reviewer’s guidance, which helped us strengthen the manuscript and enhance the clarity of its scientific contributions.

We have rewritten the “Future Research Directions”:

**Future Research Directions**

The quantitative results obtained from the Box–Wilson model open several potential directions for future research. First, the model may be extended by incorporating additional geometric and mechanical variables suggested by Buckingham’s π-theorem to provide a more comprehensive description of the behavior of threaded joints. Second, the statistically significant regression coefficients offer a solid basis for implementing reliability-based design optimization (RBDO) to control the external load factor χ under uncertain operating conditions. Furthermore, a standardized laboratory benchmark for load testing should be developed to evaluate various joint configurations and validate the model’s applicability on a broader scale. These directions arise directly from the core scientific contributions of the present study.
